# Increased urinary adiponectin level is associated with contrast-induced nephropathy in patients undergoing elective percutaneous coronary intervention

**DOI:** 10.1186/s12872-019-1143-y

**Published:** 2019-07-03

**Authors:** Jun-yi Zhang, Qiong Wang, Ru-tao Wang, Fei Li, He-xiang Cheng, Kun Lian, Yi Liu, Ling Tao

**Affiliations:** Department of Cardiology, Xijing Hospital, Fourth Military Medical University, 15 Changle West Road, Xi’an, 710032 Shannxi China

**Keywords:** Percutaneous coronary intervention, Urinary adiponectin, Contrast-induced nephropathy

## Abstract

**Background:**

Contrast-induced nephropathy (CIN) is one of major and serious complications in patients undergoing percutaneous coronary intervention (PCI). It is unknown whether increased urinary adiponectin (UAPN), a sensitive marker for early renal function impairment, is associated with an increased risk of CIN. Therefore, we prospectively investigate the association of UAPN with CIN.

**Methods:**

We prospectively enrolled 208 patients who were undergoing elective PCI. The baseline UAPN was assessed prior to PCI. The ROC analysis was used to evaluate the predictive value of UAPN for CIN. Multivariate logistic regression analysis was performed to analyze the independent risk factors for CIN.

**Results:**

Of 208 patients, CIN occurred in 19 patients (9.13%), and 6 of them (2.88%) required dialysis. Patients with CIN had a higher UAPN level than those without CIN (17.15 ± 12.36 vs. 10.29 ± 3.04 ng/ml, *P* < 0.01). ROC analysis showed that the optimal cutoff value of UAPN for predicting CIN was 12.24 ng/ml with 68.42% sensitivity and 76.72% specificity (AUC = 0.7204; 95% CI, 0.582–0.859; 푃< 0.01). Multivariate analysis demonstrated that UAPN (OR, 5.071; 95% CI,1.711–15.028; *P* < 0.01) and serum creatinine (Scr) > 124 μmol/L (OR, 4.210; 95% CI, 1.297–13.669; *P* < 0.01) were independently associated with CIN.

**Conclusions:**

Our present study showed that a higher baseline UAPN (≥12.24 ng/ml) level was significantly associated with an increased risk for developing CIN post PCI.

## Background

Contrast-induced nephropathy (CIN) is a frequent and serious complications in patients who undergo percutaneous coronary intervention (PCI), accounting for 12% hospital-acquired acute kidney injury [1]. CIN increases risk of dialysis and in-hospital mortality in patient undergoing PCI [2]. CIN is defined as “an absolute (≥0.5 mg/dl) or relative increase (≥25%) in serum creatinine (Scr) at 48-72 h after exposure to a contrast agent compared to baseline Scr values, when alternative explanations for renal impairment have been excluded” [3, 4]. Although Scr has been widely used to evaluate CIN, there are many criticisms on this biomarker, such as poor correlation to eGFR [5], and its failure to reflect early or slight changes in renal function [6]. Therefore, the search for more rapid and sensitive biomarkers in addition to traditional renal function assessment might help to identify the patients at increased risk for developing CIN.

The pathogenesis of CIN is uncertain and controversial. Endothelial dysfunction, distribution of renal blood flow and oxidative stress are considered to be possible pathophysiological mechanisms responsible for CIN [7]. Adiponectin, an adipocytokines, possesses potential anti-inflammatory property on endothelial cells by alleviating vascular inflammation [8]. Experimental model demonstrates that adiponectin deficiency mice exhibits increased albuminuria and fusion of podocyte foot processes, and adiponectin supplementation reduces podocyte permeability to albumin and podocyte dysfunction by inhibition of AMPK-NADPH oxidase signaling, which indicates that adiponectin regulates albuminuria by modulating oxidant stress [9]. A recent clinical study demonstrates that the concentration of urinary adiponectin (UAPN) is significantly enhanced in diabetes [10]. The increased UAPN is also a strong independent predictor of diabetic nephropathy progression from macroalbuminuria to end-stage renal disease and is an even better predictor than albumin excretion rate or as good as estimated glomerular filtration rate [11]. Therefore, the UAPN may be a more rapid, reliable and sensitive predictor of vascular and kidney injury, and may precede the onset of increased Scr or albuminuria.

However, no relevant report is currently available regarding whether UAPN is associated with CIN after PCI. In the present study, we aimed to investigate the association of UAPN, a sensitive marker for early vascular and kidney injury, with CIN.

## Methods

### Study population

This observational study was performed at Xijing Hospital, China, from February 2011 to May 2012. Two hundred eight adult patients with coronary heart disease who agreed to receive elective PCI treatment were enrolled prospectively. The exclusion criteria included pregnancy, infectious or inflammatory diseases, end stage renal disease, severe left heart dysfunction (NYHA≥III or LVEF<20%), allergy to contrast agent, malignant tumor, severe hepatic dysfunction and previous myocardial infarction. The study was approved by ethics committee of Xijing Hospital, China.

### Study protocol and definition

Baseline UAPN levels and Scr were tested 24 h before angiography. The midstream morning urine was collected and tested by Enzyme linked immunosorbent assay (ELISA). The UAPN value was measured as ng/ml. Regular Scr test during 48–72 h following PCI was performed to diagnose CIN. CIN is defined as “an absolute (≥0.5 mg/dl )or relative increase (≥25%) in Scr at 48-72 h after exposure to a contrast agent compared to baseline Scr values, when alternative explanations for renal impairment have been excluded” [12]. The estimated glomerular filtration rate (eGFR) was calculated by the Modification of Diet in Renal Disease (MDRD) formula, and eGFR < 60 ml/min/1.73 m^2^ was defined as impaired renal function [13].

### Coronary interventions and medications

PCI were performed according to present guideline [14]. The choice of contrast medium was left to the operator’s discretion. After PCI, all patients received the dual anti-platelet therapy (DAPT) according to present guideline [14]. Use of other medications (훽-receptor blockers, statin or angiotensin-converting enzyme inhibitor) was left to the discretion of individual cardiologist.

### Statistical analysis

Data was analyzed using the Statistical Analysis System (SAS) version 9.4for Windows. Continuous variables were expressed as mean ± SD and analyzed by Student’s t-tests or Wilcoxon rank-sum test. The categorical variables were expressed as percentages and analyzed by chi-square test or Fisher’s exact test. The receiver operating characteristics (ROC) curve was conducted to evaluate predictive value of UAPN for CIN and determine the cutoff value. The areas under the ROC curve (AUC) were calculated for each predictor as well. The statistical significance of differences after logistic regression between AUCs was also determined. Statistical analysis was conducted by logistic regression, analyzing diagnosis value of single and combined prediction of UAPN and Scr for CIN. Univariate and multivariate logistic regressions were used to identify the independent risk factors associated with CIN. In all tests, the differences were considered statistically significant at *P* < 0.05.

## Results

### Clinical characteristics

Two hundred eight eligible patients were enrolled in the present study. 19 (9.13%) of them developed CIN after undergoing PCI and 6(2.88%) required dialysis. But no CIN related death was identified. The baseline clinical characteristics were shown in Table 1. The mean age was 61.34 ± 9.87 years, and 79.33% were male. 35.10% of patients had diabetes mellitus. Hypertension and dyslipidemia were observed in 64.90 and 44.71% of patients, respectively. Compared to non-CIN patients, Patients with CIN had a higher prevalence of diabetes (57.90% vs. 32.80%, *P* = 0.03). However, there were no significant differences between the two groups regarding the age, gender, BMI, smoking status, hypertension, dyslipidemia, medication therapies prior to PCI, left ventricular ejection fraction (LVEF), contrast material use and medications.

**Table 1:** Baseline clinical characteristics of the study patients with and without CIN

Characteristics	Patients without CIN (*n* = 189)	Patients with CIN (*n* = 19)	*P* value
Age, y	61.32 ± 9.83	61.53 ± 910.51	0.93
≥70 years n (%)	35(18.52%)	5(26.32%)	0.61
Male n (%)	149(78.84%)	16(84.21%)	0.80
hypertension n (%)	126(66.67%)	9(47.37%)	0.09
Diabetes mellitus n (%)	62(32.80%)	11(57.90%)	0.03
Dyslipidemia n (%)	85(44.97%)	8(42.11%)	0.81
Smoker n (%)	118(62.44%)	8(42.11%)	0.08
Waist-to-hip ratio	0.97 ± 0.04	1.02 ± 0.14	0.27
3-vessel CAD	104(55.01%)	14(73.68%)	0.12
Contrast(ml)	229.28 ± 115.14	295.79 ± 169.91	0.07
≥300 ml n (%)	41(21.69%)	8(42.11%)	0.09
LVEF (%)	53.13 ± 8.85	50.58 ± 7.85	0.07
≤45% n (%)	32(16.93%)	7(36.84%)	0.06
Medications pre-PCI			
ACEI/ARB n (%)	106(56.08%)	8(42.11%)	0.24
Statin n (%)	50(26.46%)	4(21.05%)	0.81
β-blocker n (%)	117(61.90%)	8(42.11%)	0.09
Medications post-PCI			
Aspirin n (%)	189(100.00%)	19(100.00%)	
Clopidogrel n (%)	189(100.00%)	19(100.00%)	
Low molecular weight heparin n (%)	189(100.00%)	19(100.00%)	
Statin n (%)	189(100.00%)	19(100.00%)	
ACEI/ARB n (%)	188(99.47%)	18(94.74%)	0.18
β-blocker n (%)	179(94.71%)	16(84.21%)	0.19

Data are mean ± standard deviation or number (%). *CAD* Coronary heart disease, *CIN* Contrast-induced nephropathy, *LVEF* Left ventricular ejection fraction, *ACEI* Angiotensin-converting enzyme inhibitor, *ARB* Angiotensin receptor blocker

### Baseline laboratory characteristics

The baseline laboratory characteristics were shown in Table 2. Patients with CIN had a higher baseline UAPN level (17.15 ± 12.36 vs.10.29 ± 3.04 ng/ml, *P* < 0.01), higher baseline fasting blood glucose(FBG)(8.06 ± 2.72 vs. 6.21 ± 2.74 mmol/L, *P* < 0.01), higher Hs-CRP level (15.42 ± 13.99 vs. 7.90 ± 14.14 mg/L, *P* < 0.01), lower eGFR (49.57 ± 19.03 vs. 64.14 ± 16.55 ml/min/1.73m^2^, *P*<0.01) than those without CIN. Additionally, the number of patients with eGFR< 60 ml/min/1.73m^2^ was significantly larger in CIN group (73.68% vs. 43.39%, *P* = 0.01), and the number of patients with Scr > 124 μmol/L was also significantly larger in CIN (36.84% vs. 6.88%, *P* < 0.01) than those in non-CIN group.

**Table 2:** Baseline laboratory data of the study patients with and without CIN

Characteristics	Patients without CIN (n = 189)	Patients with CIN (n = 19)	*P* value
TC (mmol/L)	3.79 ± 0.93	3.82 ± 0.88	0.97
TG (mmol/L)	1.67 ± 1.09	1.51 ± 0.82	0.68
HDL (mmol/L)	0.95 ± 0.29	0.84 ± 0.21	0.19
LDL-C(mmol/L)	2.35 ± 0.84	2.49 ± 0.95	0.75
FBG (mmol/L)	6.21 ± 2.74	8.06 ± 2.72	< 0.01
Scr (μmol/L)	88.82 ± 21.13	119.89 ± 40.81	<0.01
> 124umol/L	13(6.88%)	7(36.84%)	< 0.01
Hs-CRP (mg/L)	7.90 ± 14.14	15.42 ± 13.99	< 0.01
Urinary albumin (g/24 h)	0.26 ± 0.57	0.47 ± 0.82	0.12
Hemoglobin (g/L)	134.24 ± 16.22	125.47 ± 20.40	0.087
UAPN (ng/ml)	10.29 ± 3.04	17.15 ± 12.36	< 0.01
eGFR(ml/min/1.73m^2^)	64.14 ± 16.55	49.57 ± 19.03	< 0.01
< 60 ml/min/1.73m^2^ n (%)	82(43.39%)	14(73.68%)	0.01

Data are mean ± standard deviation. *TG* Triglyceride, *TC* Total cholesterol, *HDL* High density lipoprotein, *LDL-C* Low-density lipoprotein cholesterol, *FBG* Fasting blood-glucose, *UAPN* Urinary adiponectin, *Scr* Serum creatinine, *Hs-CRP* High-sensitivity C-reactive protein, *eGFR* Estimated glomerular filtration rate, *CIN* Contrast-induced nephropathy

### Role of UAPN on predicting CIN

**Fig. 1:**
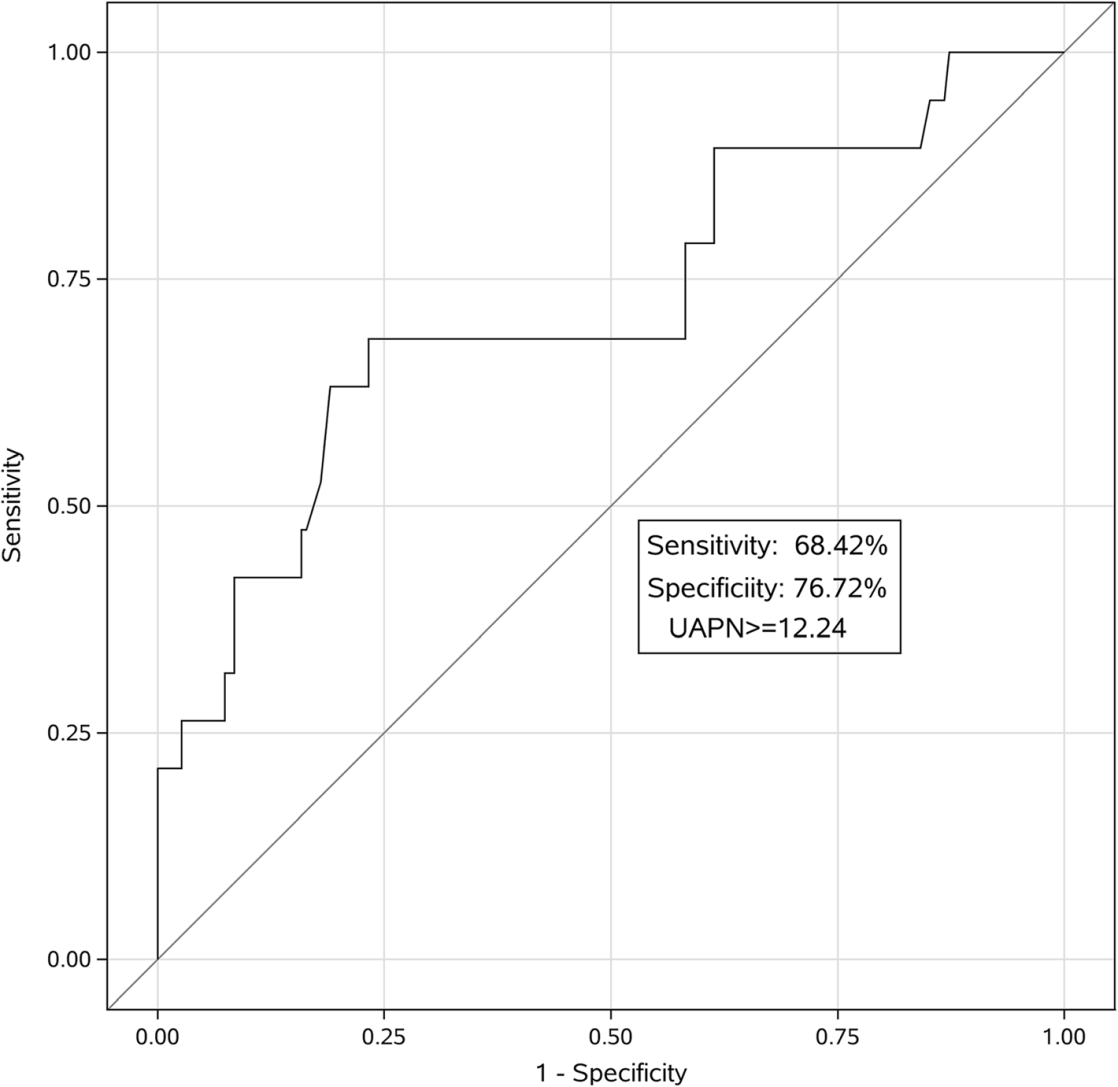
ROC curve of UAPN. ROC curve analysis demonstrated that a UAPN cutoff value of 12.24 was optimal and exhibited 68.42% sensitivity and 76.72% for specificity detecting CIN. The C-statistic was 0.7204 (95% CI, 0.582–0.859; *P*< 0.01). ROC = receiver operator characteristic; UAPN = urinary adiponectin; CIN = contrast-induced nephropathy

**Fig. 2:**
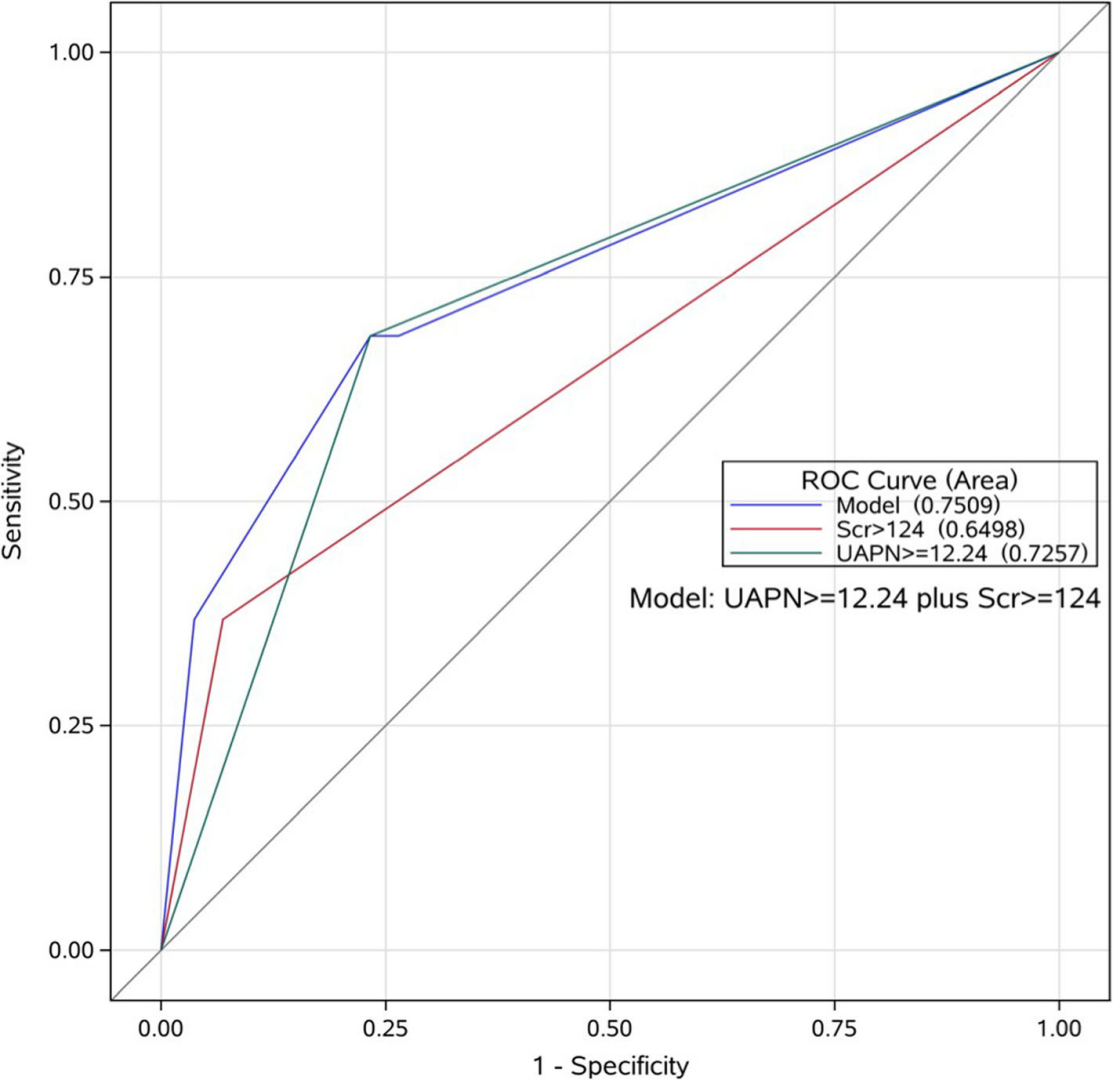
ROC curves of APN ≥ 12.24, Scr ≥ 124 and APN ≥ 12.24 plus Scr ≥ 124 for predicting CIN**.** AUC of UAPN > 12.24 μmol/L was 0.726 (95%CI: 0.614–0.837, *P* < 0.01), AUC of Scr > 124 μmol/l was 0.650 (95%CI: 0.537–0.763, *P* < 0.01), and AUC of UAPN > 12.24 μmol/L plus Scr > 124 μmol/L was 0.751 (95%CI: 0.626–0.876 *P* < 0.01). ROC = receiver operator characteristic; AUC = area under the curve; UAPN = urinary adiponectin; Scr = serum creatinine; Scr = serum creatinine

The ROC analysis was conducted to evaluate predictive value of UAPN for CIN and determine the optimal cutoff value of UAPN levels. As shown in Fig. 1, ROC analysis revealed that a UAPN cutoff value of 12.24 was optimal with 68.42% sensitivity and 76.72% specificity for detecting CIN. The area under the curve (AUC) was 0.7204 (95% CI, 0.582–0.859; *P* < 0.01). Evidence exists that patients with a Scr level > 124 μmol/l have a high incidence of CIN [15]. In the present study, the cutoff value of UAPN > 12.24 ng/ml and Scr > 124 μmol/L were used to compare their predictive value for CIN. As shown in Fig. 2 and Table 3, The AUC of UAPN > 12.24 ng/ml, Scr > 124 μmol/L and UAPN > 12.24 ng/ml plus Scr > 124 μmol/L were 0.7257 (95%CI: 0.614–0.837, *P* < 0.01), 0.6498 (95%CI: 0.537–0.763, *P* < 0.01) and 0.7509 (95%CI: 0.626–0.876 *P* < 0.01), respectively. UAPN > 12.24 ng/ml and Scr > 124 μmol/L had the same performance in predicting CIN than Scr (*P* = 0.18), whereas adding UAPN to Scr could provide a better predictive value than Scr alone(*P* = 0.04).

**Table 3:** AUC of variables for predicting CIN

Variables	AUC	95%CI	*P* value
UAPN	0.7204	0.582–0.859	*P* < 0.01
UAPN≥12.24^a^	0.7257	0.614–0.837	*P* < 0.01
Scr ≥ 124	0.6498	0.537–0.763	*P* < 0.01
UAPN≥12.24 plus Scr ≥ 124^b^	0.7509	0.626–0.876	*P* < 0.01

^a^UAPN ≥ 12.24 and Scr ≥ 124 have the same performance in predicting CIN(*P* = 0.18)

^b^Adding UAPN≥12.24 to Scr ≥ 124 could provide a better predictive value than Scr ≥ 124 alone (*P* = 0.04). *AUC* Area under the curve, *UAPN* Urinary adiponectin, *Scr* Serum creatinine, *CIN* Contrast-induced nephropathy

Univariate and multivariate logistic regressions were used to identify the independent risk factors associated with CIN. As shown in Table 3, Univariate logistic regression found that diabetes mellitus, Scr > 124 μmol/L and UAPN> 12.24 ng/ml were associated with CIN (all *P* < 0.05). The contrast volume > 300 ml and Hs-CRP as common risk factor used in clinical practice were also included in the multivariate logistic regression analysis [16, 17]. Multivariate analysis indicated that UAPN> 12.24um/ml (OR, 5.071; 95% CI, 1.711–15.028; *P* < 0.01) and Scr > 124 μmol/L (OR, 4.210; 95% CI, 1.297–13.669; *P* < 0.01) remained significant predictors for developing CIN in patients undergoing elective PCI (Table 4).

**Table 4:** Univariate and multivariate logistic regression analyses for CIN

Risk Factor	Univariate logistic regression			Multivariate logistic regression		
	*P* value	OR	95% CI	*P* value	OR	95% CI
Diabetes Mellitus	0.04	2.817	1.078–7.356			
Hs-CRP	0.05	1.022	1.000–1.046			
Scr > 124 μmol/L	< 0.01	7.897	2.658–23.469	< 0.01	4.210	1.297–13.669
Contrast volume > 300 ml	0.05	2.625	0.991–6.955			
UAPN> 12.24 μm/ml	< 0.01	7.140	2.563–19.890	0.01	5.071	1.711–15.028

Multiple logistic regression model was conducted using stepwise method. *Scr* Serum creatinine, *Hs-CRP* High-sensitivity C-reactive protein, *UAPN* Urinary adiponectin

## Discussion

In the present study, we investigated the predictive value of UAPN for the risk of CIN in patients undergoing PCI. For the first time, we demonstrated that patients with high baseline UAPN (≥12.24 ng/ml) are at a significantly higher risk for developing CIN post PCI. adding UAPN to Scr could provide a better predictive value than Scr alone. In addition to traditional renal function assessment, UAPN levels, non-invasive biomarker, could be useful in selecting patients at increased risk CIN that requires closer monitoring post PCI.

It is reported that the prevalence of CIN in patients undergoing PCI ranged from 2 to 25% [18].Patients who develop CIN after PCI have prolonged hospitalization, increased costs, increased rates of end-stage renal failure, myocardial infarction, repeat revascularization [19]. More importantly, patients who require dialysis after developing CIN have a 40% in-hospital mortality and 80% 2-year mortality rates [20]. In the present study, 9% of patients developed CIN, which was consistent with previous reports [15]. 6(2.88%) required dialysis. But no CIN related death was identified.

The pathophysiology of CIN is still controversial. Evidence exists that endothelial dysfunction and oxidative stress may be responsible for the CIN [7, 21, 22]. The intervention strategy for treatment of CIN are limited and the pre-operative prevention is of most importance. Therefore, it is important to identify high-risk patients who are susceptible to CIN before PCI. Although Scr has been widely used to evaluate CIN, there are many criticisms on this biomarker, such as poor correlation to eGFR [5], and its failure to reflect early or slight changes in renal function [6]. Therefore, the search for more rapid, reliable, and sensitive biomarkers in addition to traditional renal function assessment might help to the diagnosis and screening of patients at high risk for developing CIN. Adiponectin, an adipocytokines, possess potential anti-inflammatory property on endothelial cells by alleviating vascular inflammation [23, 24]. Experimental model demonstrated that adiponectin deficiency mice exhibited increased albuminuria and fusion of podocyte foot processes, and adiponectin supplementation could reduce podocyte permeability to albumin and podocyte dysfunction by inhibition of AMPK-NADPH oxidase signaling, which indicates that adiponectin could regulate albuminuria by modulating oxidant stress [9]. Existing evidence also demonstrates that the accumulation of adiponectin is significantly increased in the injured kidney, which prevents glomerular injury by inhibiting oxidative stress and inflammation [25]. A recent clinical study demonstrated that the concentration of urinary adiponectin (UAPN) is significantly enhanced in diabetes [10]. The increased UAPN is also a strong independent predictor of diabetic nephropathy progression from macro-albuminuria to end-stage renal disease and was an even better predictor than albumin excretion rate or as good as estimated glomerular filtration rate [11].Therefore, the increased UAPN may be a reliable and sensitive biomarker in addition to traditional renal function for predicting CIN. In the present study, we firstly reported that the patients with CIN post PCI had a significant increase of baseline UAPN level. Multivariate analysis confirmed that UAPN concentrations remained independently predictor of CIN after adjusted for diabetes mellitus, Scr, contrast use and hs-CRP. More importantly, adding UAPN to Scr could provide a better predictive value than Scr alone. Therefore, in addition to traditional renal function assessment, UAPN levels, a non-invasive biomarker, could be useful in selecting patients at increased risk CIN that requires closer monitoring post PCI.

Except for UAPN, our multivariate logistic regressions model showed that Scr > 124umol/L is also an independent risk factor for CIN, which is consistent with previous study [15]. Existing evidence has demonstrated that old age, hypertension, diabetes mellitus, hs-CRP, eGFR< 60 ml/min/1.73m^2^, high contrast dose et al. [18] are also predictors for CIN in the patient undergoing PCI. In the present study, although we found that patients in CIN group had significantly higher baseline FBG, hs-CRP and lower eGFR than those in non-CIN group, we did not find the predictive value of above-mentioned predictors in multivariate logistic regressions model. This may be attributed to the differences of the study cohort and sample size.

### Limitation

Our present study has some limitations. Firstly, this study is an observational study and conducted in a single center. Secondly, this study included a relatively small sample size. The predictive value of UAPN for CIN should be validated in a larger population. Thirdly, the UAPN level was measured only once at admission, without correction for potential variability in levels. Fourthly, previous studies have shown that the high molecular weight(HMW)APN is the main APN isoform, whereas the low molecular weight (LMW) isoform is also present in the urine of patients with diabetes [10, 11]. But we were not able to measure the UAPN isoforms, which may be more predictive for CIN.

## Conclusion

Our present study showed that a higher baseline UAPN (≥12.24 ng/ml) level was significantly associated with an increased risk for developing CIN post PCI. The combination of UAPN with Scr showed a better performance than Scr alone. As a novel and easy-to-obtain method, in addition to renal function assessment, measurement of UAPN may emerge for the clinical assessment of CIN in patients undergoing PCI. The predictive of UAPN for CIN need to be validated in a larger population.

## Data Availability

The datasets used and analyzed during the current study are available from the corresponding author on reasonable request.

## Abbreviations

APT: Dual anti-platelet therapy
AUC: Area under the curve
CIN: Contrast-induced nephropathy
LVEF: Left ventricular ejection fraction
PCI: Percutaneous coronary intervention
ROC: Receiver operating characteristic
UAPN: Urinary adiponectin
